# MicroRNA-495-3p inhibits multidrug resistance by modulating autophagy through GRP78/mTOR axis in gastric cancer

**DOI:** 10.1038/s41419-018-0950-x

**Published:** 2018-10-19

**Authors:** Sheng Chen, Jian Wu, Kai Jiao, Qiong Wu, Jiaojiao Ma, Di Chen, Jianqin Kang, Guodong Zhao, Yongquan Shi, Daiming Fan, Guohong Zhao

**Affiliations:** 1 State key Laboratory of Cancer Biology, National Clinical Research Center for Digestive Diseases and Xijing Hospital of Digestive Diseases, Fourth Military Medical University, Xi’an, China; 2 Department of Endocrinology, Tangdu Hospital, Fourth Military Medical University, 710038 Xi’an, Shanxi China; 3 Department of Pediatrics, Tangdu Hospital, Fourth Military Medical University, 710038 Xi’an, Shanxi China; 4 Gloria Gene Biotechnology Co. Ltd, 200120 Shanghai, China

## Abstract

Multidrug resistance (MDR) accounts for poor prognosis in gastric cancer (GC). MicroRNAs (miRNAs) are critical regulators of MDR via modulation of the target genes. The present study revealed that miR-495-3p could act via a target gene, *GRP78*, to regulate the process of autophagy and inhibit MDR. Based on the in vitro and in vivo gain-of-function or loss-of-function experiments, overexpression of miR-495-3p was sufficient to reverse the MDR to four chemotherapeutics in vitro and inhibit the tumor growth in vivo. Moreover, GRP78 was positively associated with the occurrence of autophagy. Thus, reducing the expression of *GRP78* by siRNA resulted in autophagy-suppressive activity similar to that of miR-495-3p on mammalian target of rapamycin (mTOR) and its substrates activation and autophagy inhibition, while restoring GRP78 attenuated the anti-autophagy effects caused by miR-495-3p. Clinically, either miR-495-3p downregulation or GRP78 upregulation was associated with malignant phenotypes in patients with GC. In conclusion, these findings demonstrate that miR-495-3p is an important regulator of autophagy balance and MDR by modulating the GRP78/mTOR axis. In addition, miR-495-3p and GRP78 could be used as prognostic factors for overall survival in GC, which implicates miR-495-3p as a therapeutic target in cancer.

## Introduction

Multidrug resistance (MDR) is a significant clinical issue with respect to chemotherapy in cancer, especially gastric cancer (GC)^1^. A variety of mechanisms underlying MDR have been surveyed including increased repair of DNA damage, increased energy-dependent efflux of hydrophobic drugs, and stress-induced alteration of genes in the microenvironment^2,3^. Several anti-MDR drugs, such as calcium channel blockers verapamil and quinidine, cyclosporin A, PSC833, and LY335979, primarily aiming at the ABC transporter P-Glycoprotein failed in clinical application owing to side-effects^4,5^. Among various mechanisms of MDR, cancer cells’ response or adaption to the stressful environment invoked by the anti-cancer agents may be a vital cause. Autophagy is an important defense mechanism of tumor cells against chemotherapeutic drugs.

Autophagy is a highly conserved catabolic process, in which, the bulk is sequestered into a double-membrane vesicle and degraded by lysosomes^6^. The damaged cellular components can be degraded to substances that support metabolism and promote further tumor cell growth and survival. When subjected to stressful conditions, cancer cells respond rapidly to maintain the metabolic homeostasis through autophagy. Some types of tumor cells maintain in a high level of basal autophagic flux. While some others gradually acquire drug resistance by increasing autophagic flux^7^. Therefore, autophagy underlies this type of acquired resistance phenotype of some cancer cells during chemotherapy. Accumulating evidences demonstrated that miRNA-regulated autophagy is involved in resistance or sensitization to chemotherapy.

MicroRNAs (miRNAs) are small, non-coding RNAs, 19–24 nucleotides in length that can regulate the target genes post-transcriptionally through complementary binding to their target mRNAs. Although the relationship among miRNA, autophagy, and anti-cancer therapy resistance is complicated and has not been well elucidated, miRNA may underlie the key aspects of chemotherapeutic resistance: miRNA can function either as a promoter or a suppressor to regulate the MDR of GCs. One of the miRNAs, miR-508-5p, can reverse the GC cell resistance to multiple chemotherapeutics in vitro and sensitize the tumors to chemotherapy in vivo^8^, while the overexpression of miR-27a could inhibit the sensitivity of drugs on GC cells^9^. miR-23b-3p shows a low-expression in GC drug-resistant cell and is a potent inhibitor of autophagy by selectively downregulating the ATG12 expression^10^. miR-30a, a member of miR-30 family, can sensitize the tumor cells to *cis*-DDP via reducing beclin-1-mediated autophagy^11^. However, excessive autophagy may lead to autophagic cell death, a form of physiological cell death which is contradictory to type I programmed cell death (apoptosis), depending on tumor types and treatment characteristics. Previous studies demonstrated that not only the expression of mir-495-3p was declined in GC samples because of hypermethylation of promoter area^12^, but also that the upregulation of mir-495 could inhibit the migration, invasion, and growth of GC cells^12,13^. However, the exact role and molecular mechanism of miR-495-3p underlying the regulation of GC MDR is yet to be elucidated.

In the present study, we found that ectopic expression of miR-495-3p can sensitize the GC cells to chemotherapy, which resulted from the inhibition of autophagy induced by miR-495-3p. Moreover, miR-495-3p was found to regulate autophagy in MDR cells by modulating the target gene *GRP78* and mammalian target of rapamycin (*mTOR*). Finally, we confirmed the inverse correlation between the expression of miR-495-3p and its target *GRP78* in GC and found that GC patients with low miR-495-3p expressions tended to have a poor prognosis.

## Result

### miR-495-3p is downregulated in GC and inhibits MDR and proliferation of GC MDR cells

**Fig. 1:**
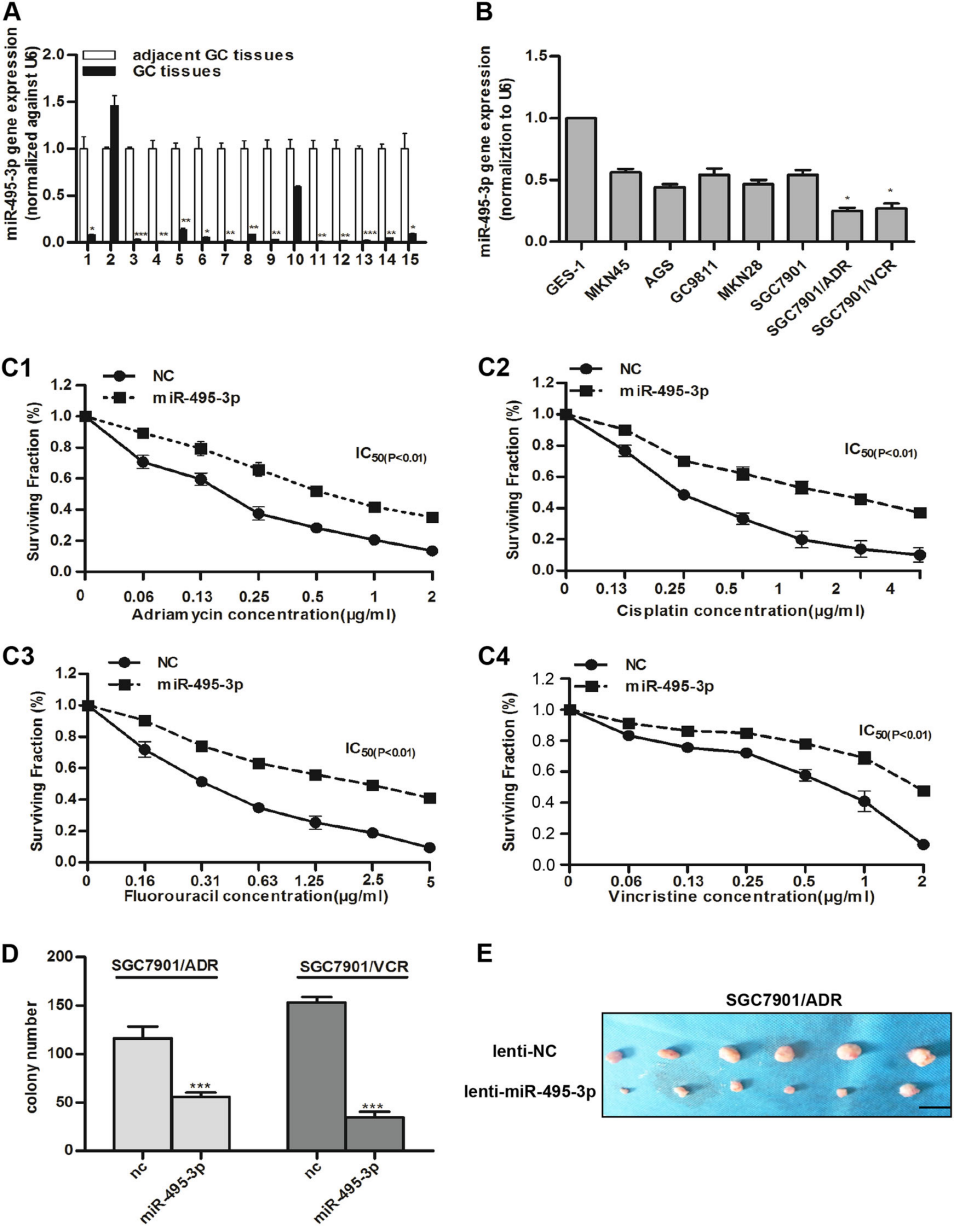
miR-495-3p is downregulated in GC and inhibits MDR and proliferation of GC MDR cells. **a** Real-time PCR detected the relative expression level of miR-495-3p in peri-tumor tissue and gastric cancer tissue in 15 paired samples. **b** The expression level of miR-495-3p in normal gastric epithelial cell line, five gastric cancer cell lines and GC MDR cells. **c** Four chemotherapeutic drugs (c1 adriamycin; c2 cisplatin; c3 fluorouracil; c4 vincristine) with indicated concentration gradient were added to the medium of SGC7901/ADR transfected with miR-495-3p mimics or nc. Then, cell survival was measured using the MTT assay. **d** Colony formation assays were performed to evaluate the proliferative functions of GC MDR cells transfected with miR-495-3p mimic or nc. 10^3^ cells per well were seeded initially in 6-well plates. **e** Representative images of tumors 4 weeks after subcutaneous injection of 10^7^ SGC7901/ADR transfected with lenti-NC or lenti-miR-495-3p into the right flank of the nude mice. (Scale bar = 1 cm). All experiments were performed in triplicates. All values were expressed as mean ± SD, *n* = 3 for each group. *P < 0.05, **P < 0.01, ***P < 0.001

miR-495 was previously reported to sensitize MDR cancer cells and downregulated in various cancers^14,15^. Therefore, whether it was also downregulated in GC compared to adjacent GC tissues is yet to be elucidated. Real-time PCR was applied to detect the expression of miR-495-3p in cancer and the corresponding adjacent GC tissues in 15 GC patients, who underwent gastrectomy at Xijing Hospital. Figure 1a demonstrated that the expression of miR-495-3p was lower in 14/15 randomly selected human GC tissues as compared to the peri-tumor tissues. Furthermore, we compared the expression of miR-495-3p in SGC7901 ADR/VCR, several gastric cancer cell lines, and normal gastric epithelial cells-1 (GES-1) and found that miR-495-3p was downregulated in GC MDR cells as compared to that in gastric cancer cell and GES-1 cells. Next, we transfected SGC7901/ADR with miR-495-3p mimic and examined the survival ability when exposing to four chemotherapy drugs with different concentration gradients using the MTT assay. Intriguingly, MDR was significantly damaged in miR-495-3p-overexpressed SGC7901/ADR (Fig. 1c1–c4). Colony formation assay (Fig. 1d) and in vivo subcutaneous tumor in BALB/C nude mice (Fig. 1e) also confirmed that miR-495-3p could arrest the proliferation ability of MDR cells in vivo and in vitro.

### miR-495-3p inhibits autophagy in GC MDR cells

As autophagy exerts a dual role that contributes to the anti-cancer efficacy of chemotherapy drugs as well as drug resistance on cancer cells^7^, we investigated whether miR-495-3p can regulate the process of autophagy in GC MDR cells. First, we determined the autophagic level in MDR cells and its parental sensitive cancer cells. The cells stained with the lipidated form of microtubule-associated protein 1 light chain 3 (LC3B), a marker of autophagy, demonstrated that MDR cells expressed excessive LC3 puncta (Supplementary Fig. 1A). Also, MDR cells expressed a high level of autophagy-associated protein and gene (Supplementary Fig. 1B and 1C) as assessed by western blotting and real-time PCR. Conversely, the levels of sequestosome 1 (P62), an indicator of cytosolic protein clearance, were decreased in MDR cells. Thus, these data implied that MDR cells might resist the chemotherapeutics through autophagy.

**Fig. 2:**
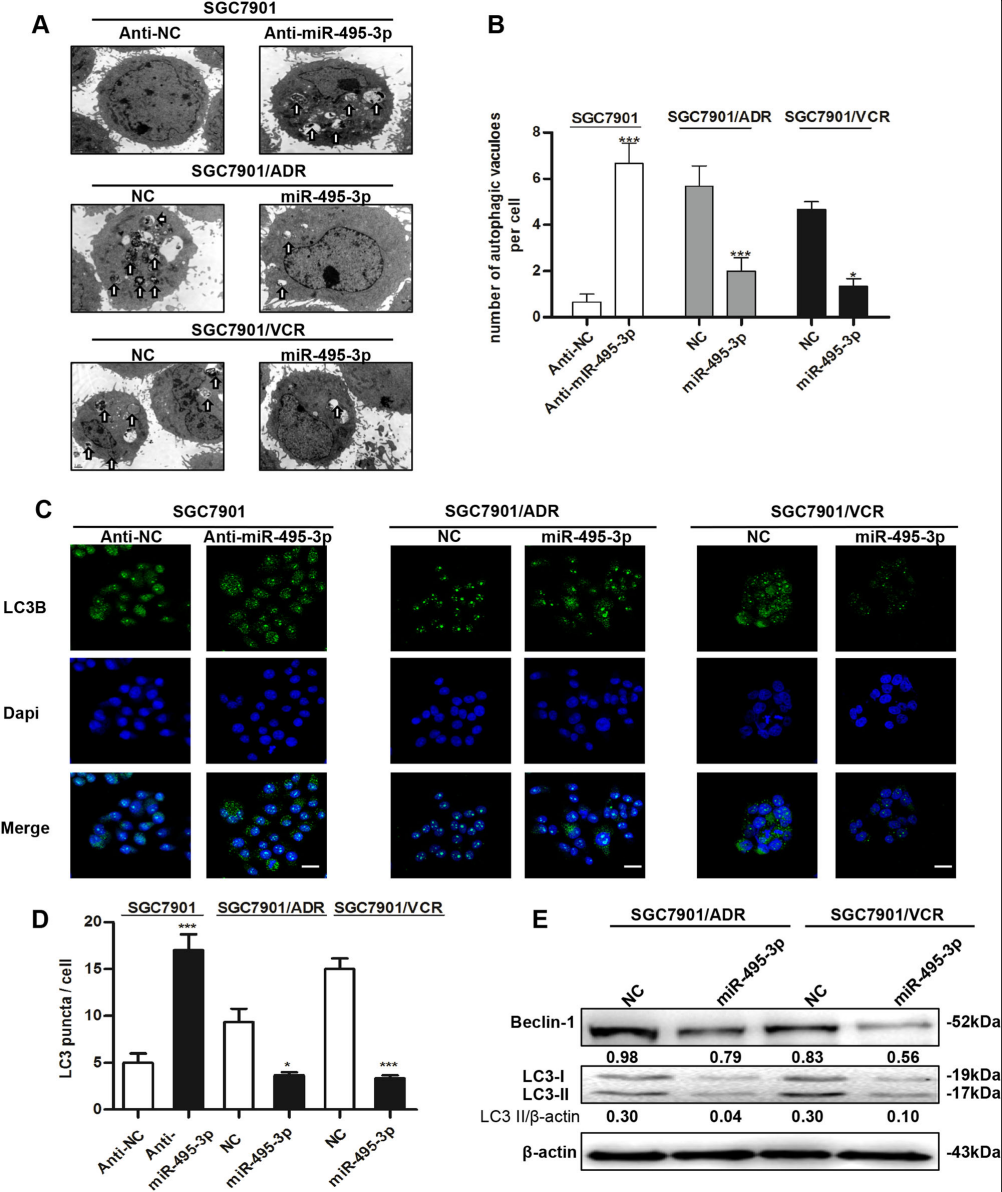
miR-495-3p inhibits autophagy in GC MDR cells. **a** Transmission electron micrographs of morphological changes in SGC7901 transfected with miR-495-3p inhibitor or GC MDR cells transfected with miR-495-3p mimic. White arrows represent autophagosomes or autolysosomes. (Scale bar = 500 nm). **b** Quantitative analysis of the number of autophagic vesicles. **c** Representative images and **d** Quantification of LC3 puncta (green) in SGC7901 transfected with miR-495-3p inhibitor or GC MDR cells transfected with miR-495-3p mimics. Scale bars: 50 μM. **e** Western blot analysis of the protein levels of Beclin-1, and LC3I/II in GC MDR cells transfected with miR-495-3p mimic or nc. All values were expressed as mean ± SD, *n* = 4 for each group. *P < 0.05, **P < 0.01, ***P < 0.001

To determine the inhibitory effect of miR-495-3p on autophagy, transmission electron microscopy was employed for the detection and quantification of autophagosomes in cellular cross-sections. SGC7901 cells were transfected with miR-495-3p inhibitor, and MDR cells were forced to overexpress miR-495-3p by the transient transfection of miR-495-3p mimic. Figure 2a and b showed that miR-495-3p inhibitor could significantly promote the formation of autophagosomes or autolysosome in SGC7901; however, miR-495-3p mimic inhibited the number of autophagosomes in GC MDR cells. Consistently, GC MDR cells with miR-495-3p overexpression had significantly lower levels of punctate LC3 as compared to the control cells (Fig. 2c, d). As expected, the results of western blotting also confirmed that miR-495-3p could reduce autophagic proteins such as beclin-1 and LC3-II (Fig. 2e). Moreover, overexpression of miR-495-3p could downregulate the expression of P-glycoprotein which serves as an efflux pump and reduces drug accumulation in cancer cells. Overexpression of miR-495-3p can also increase MDR cells’ level of apoptosis through upregulating Bax and downregulating Bcl-2 (Supplementary Fig 2). Thus, our data hypothesized that autophagy was impaired by upregulation of miR-495-3p in MDR cells.

### miR-495-3p directly targets GRP78 at the post-transcriptional level

**Fig. 3:**
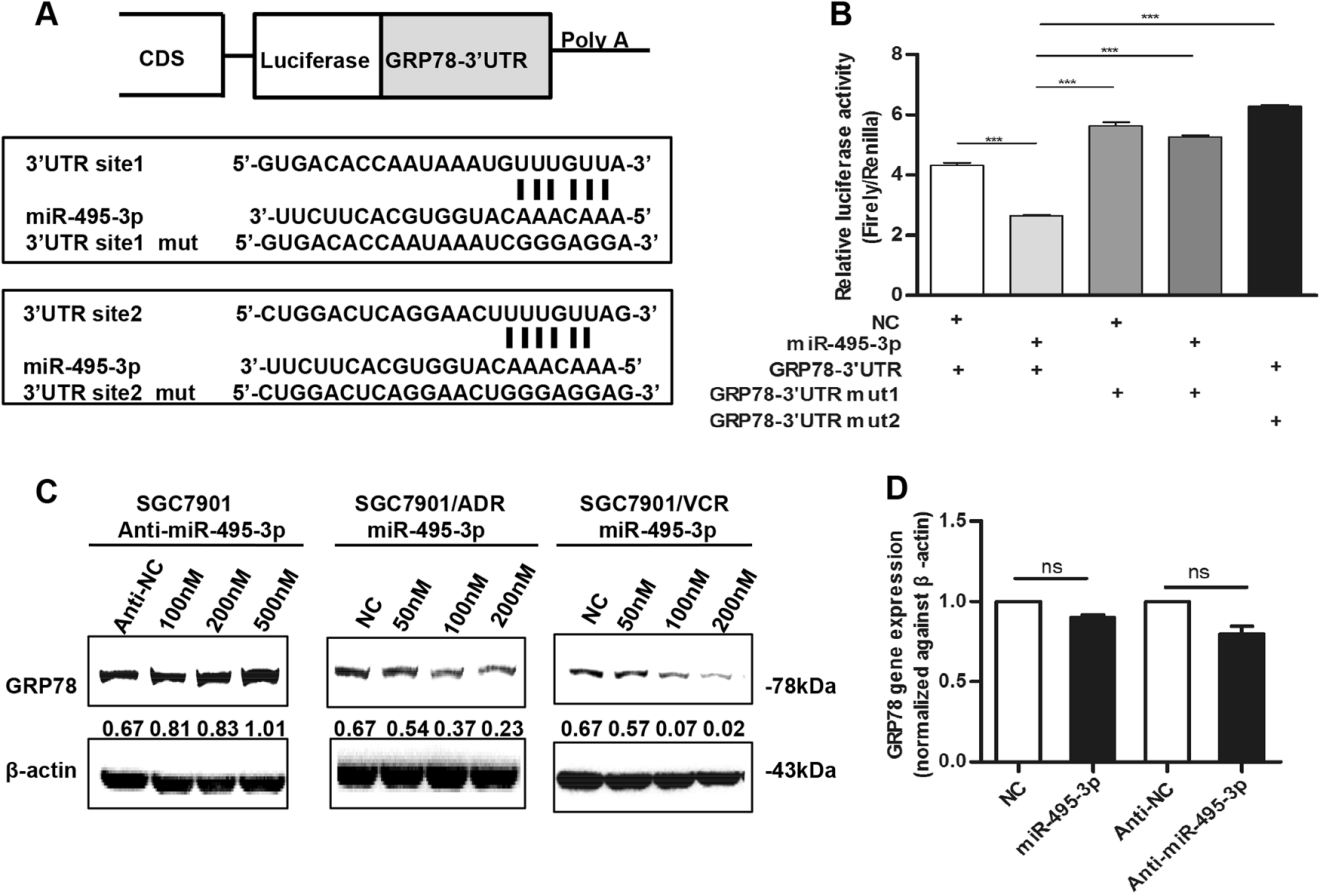
miR-495-3p directly targets GRP78 at the post-transcriptional level. **a** Illustration of the GRP78 3′-UTR-containing reporter constructs. Mutations were generated at two predicted miR-495-3p binding sites located in the GRP78 3′-UTR. **b** Representative luciferase activity in SGC7901 cells co-transfected with wild-type or mutated reporter plasmids and nc, miR-495-3p mimic. **c** Western blot showed the changes in GRP78 protein levels after transient transfection of miR-495-3p inhibitor or mimic with indicated concentration gradient as compared to the negative controls (nc or anti-nc, respectively). **d** Real-time PCR determined the GRP78 level in SGC7901 after transfection with miR-495-3p inhibitor/mimic or anti-nc/nc. NS is no significance. All values were expressed as mean ± SD, *n* = 3 for each group. *P < 0.05, **P < 0.01, ***P < 0.001

To understand the molecular mechanism underlying the miR-495-3p-mediated suppression of MDR in gastric cancer, we searched for miR-495-3p targets using three computational methods including MiRanda, Targetscan, and RNAhybrid and identified a dozen of candidate genes that were commonly predicted as putative targets of miR-495-3p (Fig. 3a). Interestingly, GRP78 was previously reported to act as a regulator of autophagy in several types of cancer^16,17^. To determine whether GRP78 is a direct target of miR-495-3p, we constructed the 3′-UTR fragments, wherein wild-type and mutant binding sites were inserted into the region immediately downstream to the reporter gene. Luciferase reporter assays showed that miR-495-3p transfection caused a remarkable decrease in luciferase activity of plasmid harboring the wild-type 3′-UTR of GRP78. Moreover, the luciferase activity did not decline sharply in the 3′-UTR that contained the mutant binding sites (Fig. 3b). Furthermore, western blotting demonstrated that overexpression of miR-495-3p significantly suppressed GRP78 expression in GC MDR cells cells and that silencing of miR-495-3p increased the expression of GRP78 in SGC7901 cells (Fig. 3c). However, real-time PCR showed that the overexpression or inhibition of miR-495-3p did not exert any effect on the *GRP78* mRNA level (Fig. 3d), thereby indicating that miR-495-3p regulated GRP78 at the post-transcriptional level. Taken together, these results suggested that miR-495-3p regulated GRP78 expression by directly targeting its 3′-UTR.

### GRP78 expression is increased in GC and associated with poor outcomes

**Fig. 4:**
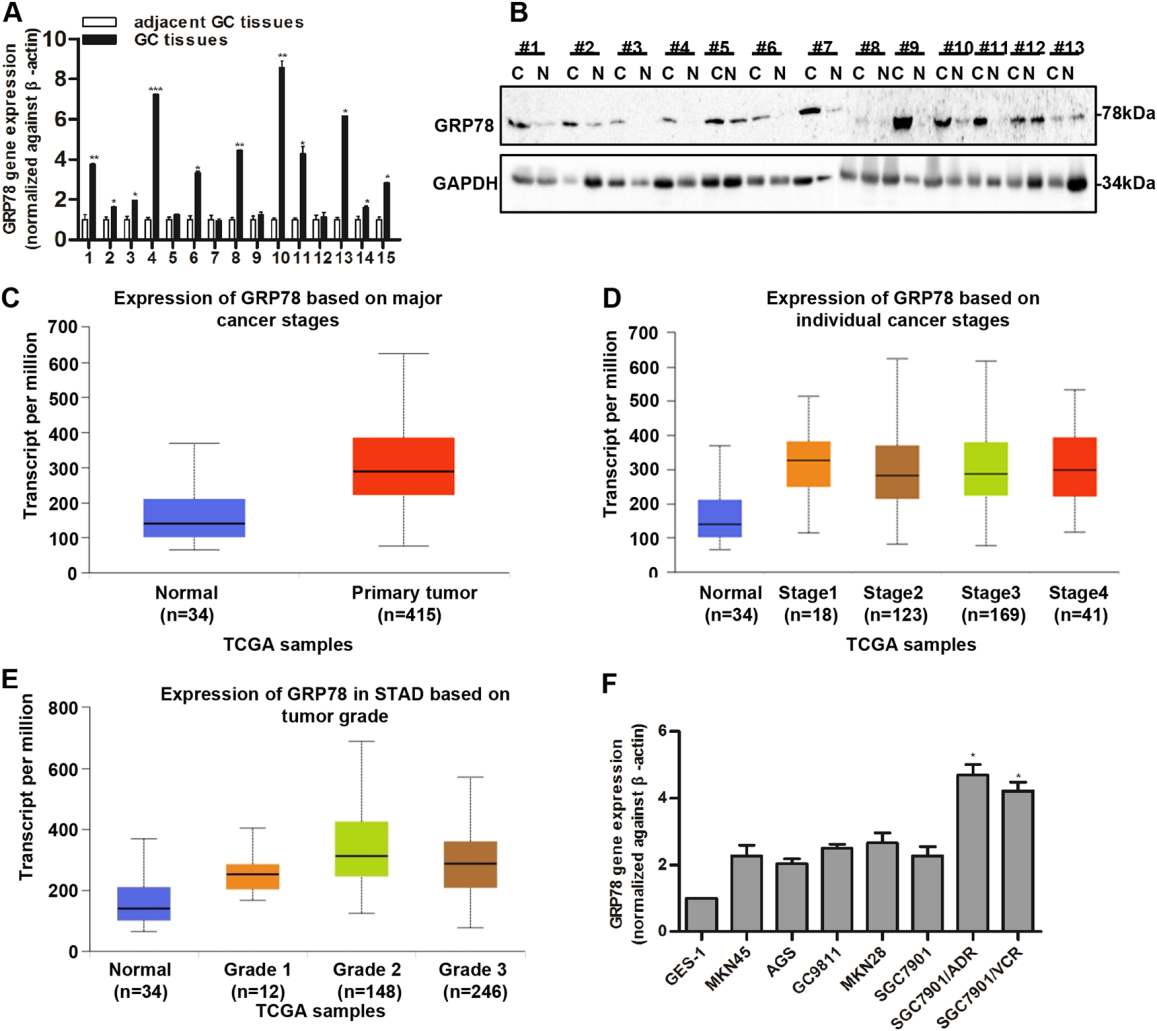
**GRP78 expression is increased and associated with poor outcomes in GC.** **a** Real-time PCR shows the expression of GRP78 in 15 paired gastric cancer samples as compared to adjacent tumor tissues. **b** Western blotting indicates the protein level of GRP78 in 13 paired gastric cancer tissues and corresponding adjacent tumor tissues (C is carcinoma, N is normal tissues). Data from TCGA show the transcript per million of GRP78 in normal and primary GC tissues (**c**) based on individual cancer stages (**d**) and tumor grades (**e**). **f** The expression of GRP78 in normal gastric epithelial cell line and five gastric cancer cell lines and GC MDR cells by Real-time PCR. All values expressed as mean ± SD, *n* = 3 for each group. *P < 0.05, **P < 0.01, ***P < 0.001

The expression of GRP78 was determined in GC fresh tissues. The results showed that the mRNA expression of *GRP78* was significantly upregulated in GC (Fig. 4a). Similarly, GRP78 protein expression was also higher in GC than the peri-tumorous tissues (Fig. 4b). Data from TCGA datasets revealed high genomic amplification of the *GRP78* gene in GC (Fig. 4c). Moreover, the genomic amplification of GRP78 was higher in individual cancer stage (Fig. 4d) and increased with tumor grade (Fig. 4e). Consistently, we measured the levels of GRP78 in primary GC cell lines, GES-1 and GC MDR cells, and found that the GRP78 expression was upregulated in GC MDR cells as compared to the GC cell lines (Fig. 4f).

### Downregulation of GRP78 by miR-495-3p inhibits autophagy in GC MDR cells

**Fig. 5:**
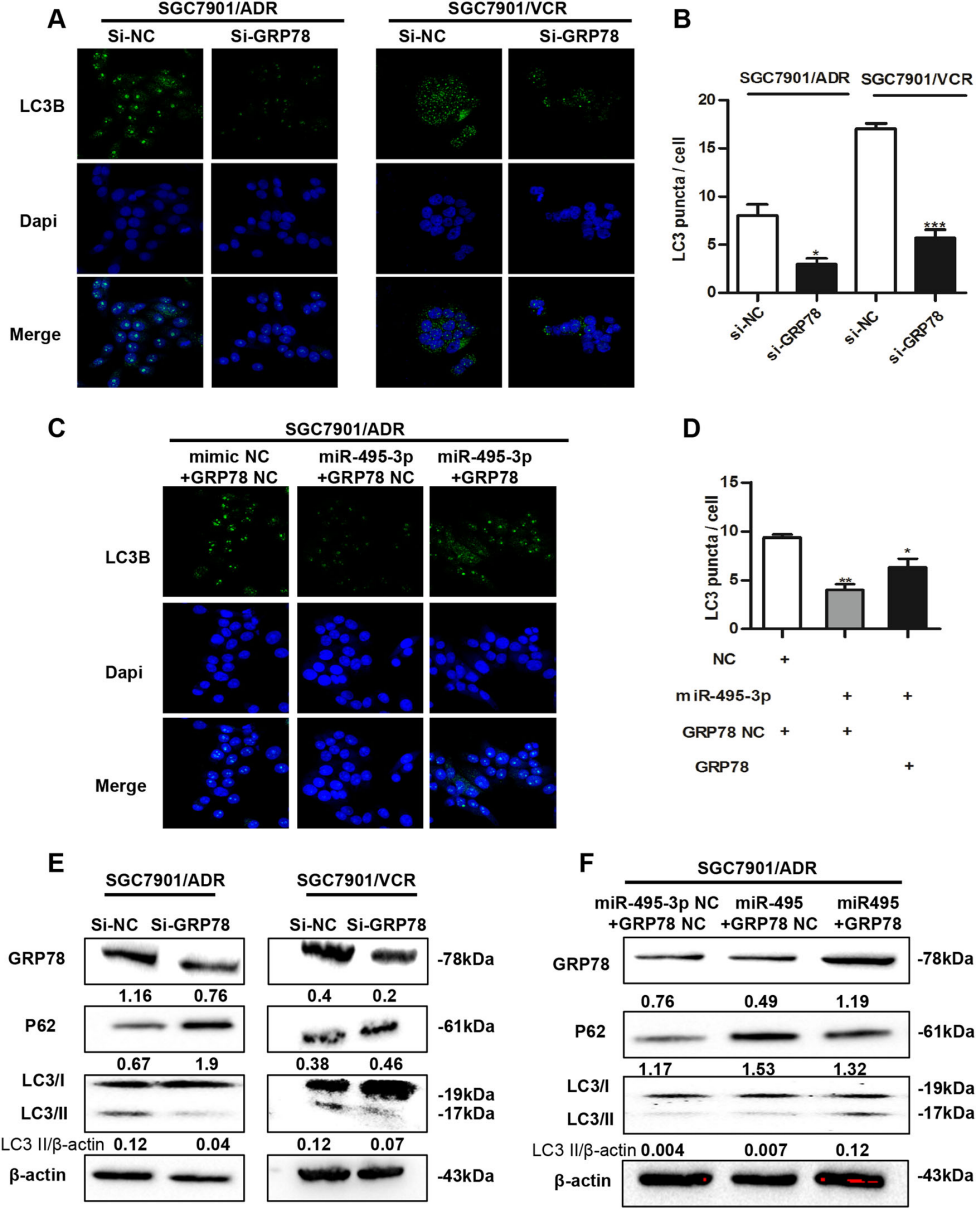
**Downregulation of GRP78 by miR-495-3p inhibits autophagy in GC MDR cells.** **a** Representative images and **b** Quantification of LC3 puncta (green) in GC MDR cells transfected with si-GRP78 or si-nc. Scale bars: 50 μM. **c** Representative images and **d** Quantification of LC3 puncta (green) in SGC7901/ADR co-transfected with GRP78 3′-UTR-mutant overexpression vector/nc and miR-495-3p/nc. **e** The expression level of GRP78, LC3BI/II, and P62 in GC MDR cells transfected with si-GRP78 or si-NC were determined by western blotting. **f** The protein level of GRP78, LC3BI/II, and P62 in SGC7901/ADR co-transfected with GRP78 3′-UTR-mutant overexpression vector/nc and miR-495-3p/nc. All values expressed as mean ± SD, *n* = 3 for each group. *P < 0.05, **P < 0.01, ***P < 0.001

To elucidate whether GRP78 is involved in MDR cell autophagy, we stained LC3B and performed western blotting to examine the levels of LC3B and p62 in GRP78-silenced cells (Fig. 5a, e). The downregulation of GRP78 decreased the number of LC3B puncta in SGC7901/ADR and VCR cells (Fig. 5a, b). Similarly, the silencing of GRP78 increased the expression of p62 and inhibited the expression of LC3B-II (Fig. 5e). To investigate whether GRP78 downregulation is essential for the suppression of autophagy by miR-495-3p, we co-transfected SGC7901/ADR cells with GRP78 3′UTR-mutant overexpression vectors or negative control vectors and miR-495-3p. As expected, the restoration of GRP78 partially blocked the miR-495-3p-mediated suppression of autophagy in MDR cells (Fig. 5c, f). As autophagy might exert a protective role against apoptosis, we also assessed the apoptosis in GRP78-silenced cells. We found that anti-apoptotic Bcl-2 decreased in GRP78-silenced cells (Supplementary Fig. 3A) and the expression of multidrug resistance protein 1 (MDR1) was also inhibited. The results of flow cytometry also revealed that the number of apoptotic cells increased in GRP78-silenced cells (Supplementary Fig. 3B). Taken together, GRP78 was identified as a major functional target of miR-495-3p that promotes autophagy in GC cells.

### miR-495-3p inhibits autophagy by activating mTOR signaling

**Fig. 6:**
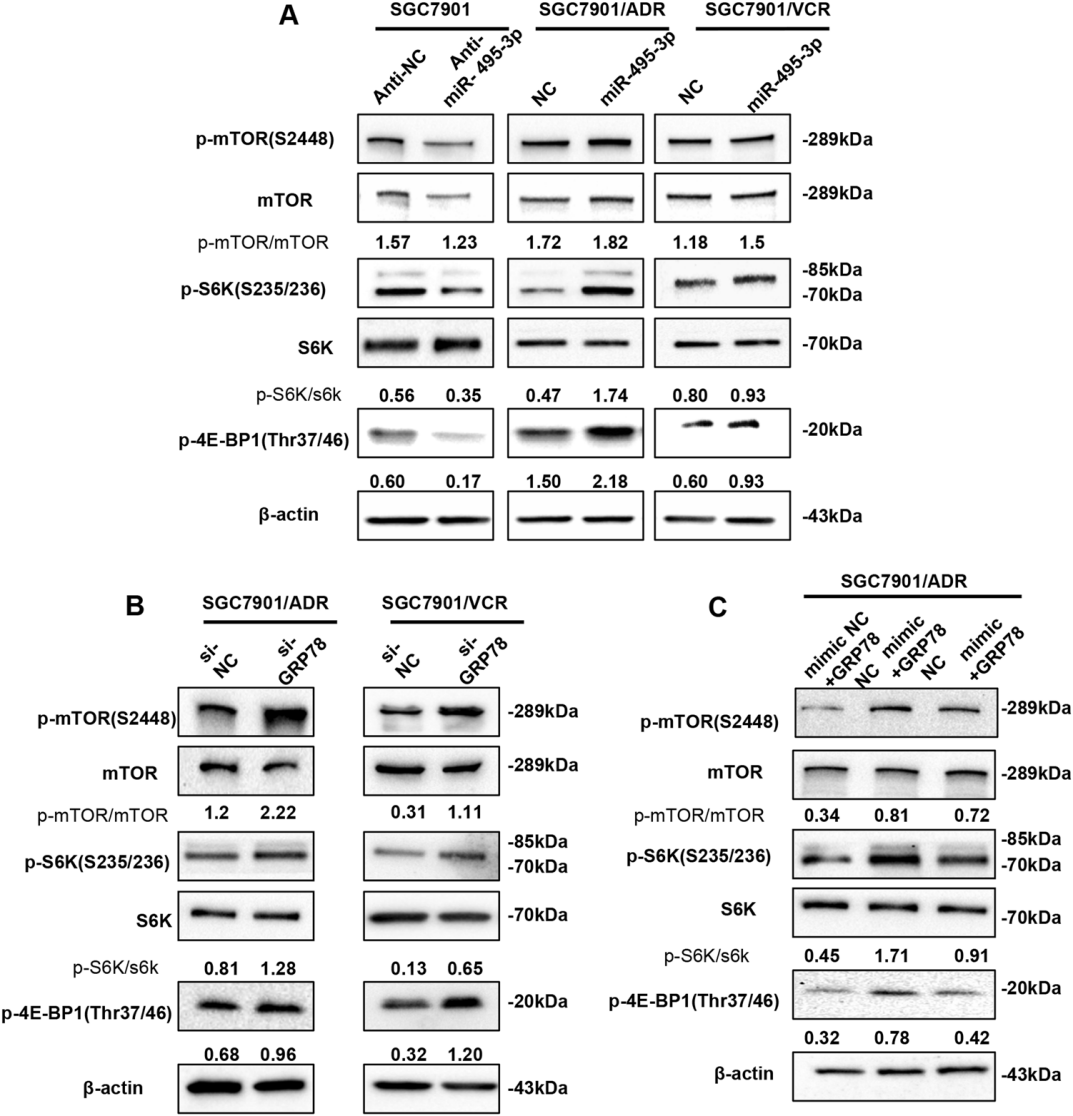
miR-495-3p inhibits autophagy by activating mTOR signaling. **a** Western blot analysis of phosphorylated mTOR (p-mTOR) and its main substrates 4E-BP1 (p-4E-BP1) and S6 (p-S6) in SGC7901 transfected with anti-miR-495-3p and GC MDR cells transfected with miR-495-3p. **b** The phosphorylation status of mTOR, S6K (p-S6K), and 4E-BP1 (p-4E-BP1) transfected with si-GRP78 and si-NC were measured by western blotting (**c**). Western blotting determines the phosphorylation of mTOR, S6K (p-S6K), and 4E-BP1 (p-4E-BP1) in SGC7901/ADR co-transfected with GRP78 3′-UTR-mutant overexpression vector/nc and miR-495-3p/nc. All values expressed as mean ± SD, *n* = 3 for each group. *P < 0.05, **P < 0.01, ***P < 0.001

As the mTOR is a well-known negative regulator of autophagy, we examined whether it was involved in the miR-495-3p-induced autophagy inhibition. Thus, we first reduced the level of miR-495-3p in SGC7901 cells and increased that in MDR cells. Subsequently, the activity of mTOR was measured by detecting the phosphorylation levels of mTOR and its substrates (4E-BP1, S6K, or S6) using western blotting (Fig. 6a). The levels of phospho-mTOR (S2448), phospho-4E-BP1 (T37/46), and phospho-S6 (S235/236) levels were increased in miR-495-3p-overexpressed GC MDR cells as compared to those in the controls, indicating that mTOR was activated. Conversely, miR-495-3p depletion in SGC7901 decreased the levels of phospho-mTOR, phospho-S6K, and phospho-S6. These data suggested that miR-495-3p regulated the activity of mTOR in MDR cells. Moreover, the mTOR pathway was markedly activated in MDR cells wherein the expression of GRP78 was suppressed, as demonstrated by the increased phosphorylation levels of mTOR, S6K, and p-4E-BP1 (Fig. 6b). Next, we restored the expression of GRP78 in SGC7901/ADR cells exhibiting miR-495-3p overexpression. Furthermore, forced expression of GRP78 decreased the phosphorylation level of p-mTOR, p-4E-BP1, and p-S6K (Fig. 6c). Thus, the current results demonstrated that upregulating GRP78 hampered the miR-495-3p-induced mTOR activation and autophagy inhibition.

### GRP78 expression inversely correlates with miR-495-3p in GC tissues

**Fig. 7:**
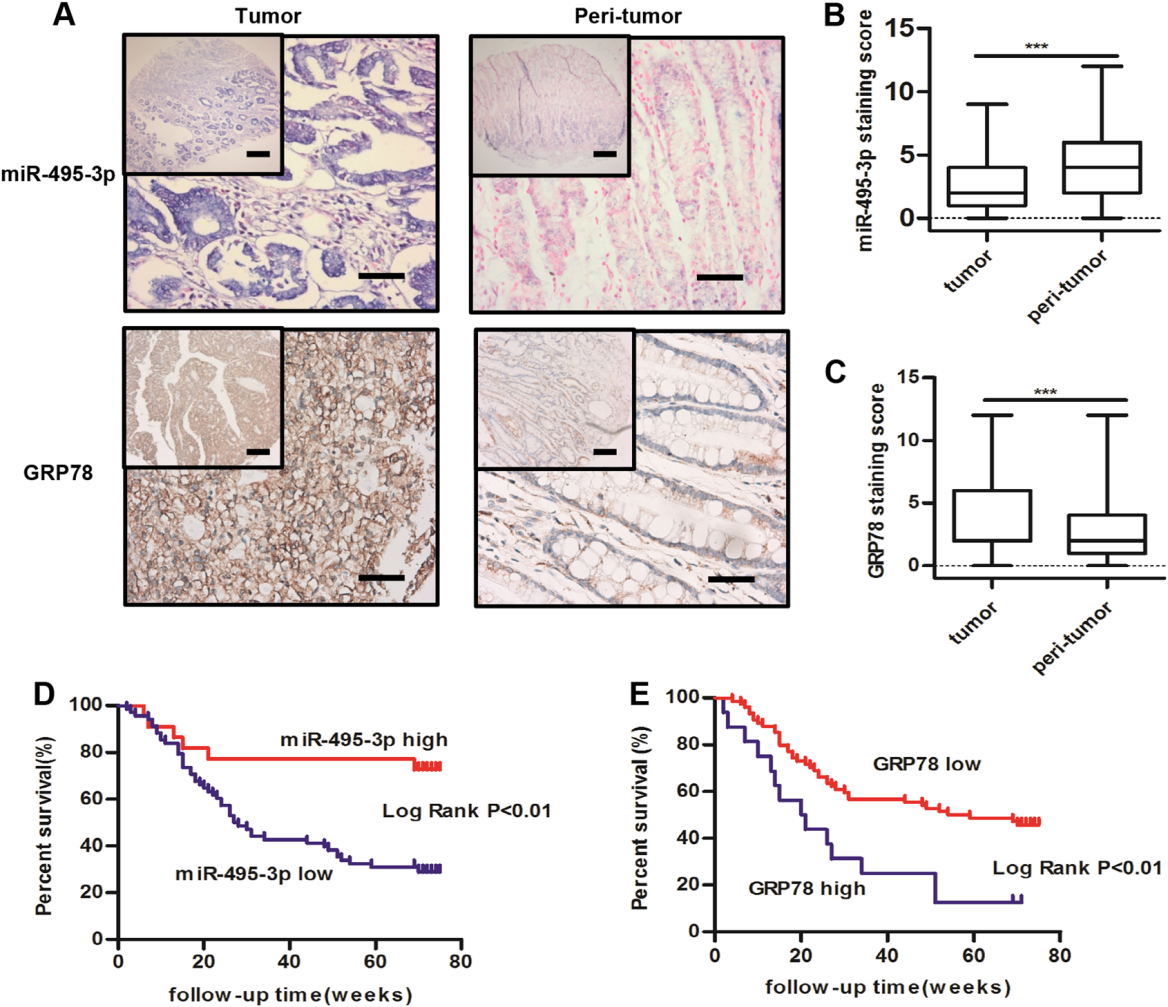
**Expression levels of miR-495-3p and GRP78 in GC specimens.** **a** The expression levels of miR-495-3p (upper) and GRP78 (lower) in peri-tumor and primary GC tissues, scale bars: 500 μm (top) and 200 μm (bottom). **b** miR-495-3p and **c** GRP78 expression staining score in peri-tumor and primary GC tissues. Kaplan–Meier survival curves of GC patients expressing miR-495-3p (**d**) and GRP78 (**e**) GC patients were ranked based on the staining score and divided into high-expression (>4) and low-expression (≤4) groups. *P < 0.05, **P < 0.01, ***P < 0.001

To examine the clinical relevance of miR-495-3p and GRP78, we measured the expression patterns of these factors using in situ hybridization and immunohistochemistry in commercial tissue microarrays, which contain 90 GC tissues and corresponding 90 peri-tumor tissues (Fig. 7a). We found that the expression levels of miR-495-3p were lower in GC, whereas the expression of GRP78 was significantly upregulated in GC (Fig. 7b, c). Moreover, the miR-495-3p expression was statistically correlated with degree of differentiation (*P* < 0.05), while that of GRP78 was statistically correlated with the tumor size and degree of differentiation (*P* < 0.05), (Table 1). Importantly, the patients in the low miR-495-3p expression group had a significantly poor prognosis than those in the high miR-495-3p expression group (Fig. 7d). Correspondingly, high GRP78 also predicted poor outcomes (Fig. 7e).

**Table 1:** Correlation of miR-495-3p and GRP78 expression with patients’ clinicopathological variables in GC tissues

Variables		miR-495-3p			GRP78		
		Low	High	*P*-value	Low	High	*P*-value
Gender							
Male		37	14	0.602	51	11	0.776
Female		18	5		23	5	
Age (year)							
≤50		7	3	0.664	10	0	0.262
>50		61	19		64	16	
Tumor size (cm)							
≤5		13	23	**0.047**	28	15	**0.011**
≥5		31	23		18	29	
TNM stage							
I + II		16	18	**0.002**	30	4	0.244
III + IV		44	12		44	12	
Degree of differentiation							
Well and moderately		7	11	**0.001**	14	4	**0.005**
Poorly		56	16		29	42	
Local invasion							
T1 + T2		9	6	0.124	13	2	0.621
T3 + T4		59	16		61	14	
Metastasis							
No		67	21	0.395	55	12	0.292
Yes		1	1		21	2	

## Discussion

MDR is defined as the resistance of cancer cells to multiple chemotherapeutic drugs with different structures and mechanisms of action, which results in relapse and metastasis in most malignant tumors^18^. Significant changes in the miRNA expression profiles were observed in the drug-resistant cancer cells as compared to the parental drug-sensitive cancer cells^19^. We selected *miR-495-3p* as a candidate gene for MDR in GC cells. Dysregulation of miR-495-3p has been reported in many cancers, including breast cancer, prostate cancer, and colorectal cancer^15,20,21^. In the present study, the precise role of miR-495-3p in regulating MDR in GC cells was studied, and the results showed that by targeting GRP78, the overexpression of miR-495-3p in GC MDR cells caused a decreased resistance to chemotherapy in vitro and vivo; therefore, it might be a potential therapeutic target for MDR treatment. To our knowledge, this study, for the first time, identified miR-495-3p as a key regulator of the chemosensitivity through regulating autophagy in GC.

Autophagy is known to have a critical role in coping with multiple forms of cellular stresses, including nutrient or growth factor deprivation, hypoxia, or damaged organelles^22,23^. Although mechanisms of MDR involves complicated and multifactorial processes, autophagy is a relatively new mechanism underlying anti-cancer drugs resistance reported recently^24^. Autophagy induced by therapeutic and metabolic stresses might exert a pro-death or pro-survival role; however, accumulating evidences suggest that the pro-survival function of autophagy plays a significant role in chemoresistance in several cancer types^25,26^. We found that MDR cells possessed a relatively higher level of autophagy as compared to their parental cell, and therefore, autophagy could be considered to participate in the process of MDR. Since mTOR is a pivotal upstream mediator of autophagy via binding and inactivation of the autophagy kinase complex ULK1/2, thereby blocking the formation of autophagosomes^27^. Our results showed that the overexpression of miR-495-3p impaired the autophagy in MDR cells both in vivo and in vitro, which was accompanied by mTOR activation and its substrates (4E-BP1, S6K, or S6). Thus, miR-495-3p inhibits MDR by regulating mTOR activity and autophagy, providing further evidence that the appropriate level of autophagy is essential for the maintenance of MDR in cancer.

The application of three miRNA target gene predicting tools identified *GRP78*, also known as HSPA5, BIP, HEL-S-89n, and MIF2 as the molecular foundation of the miR-495-3p axis. Currently, it has been demonstrated that GRP78-induced ER (endoplasmic reticulum) stress and the subsequent UPR (unfolded protein response) play critical roles in mediating proliferation, carcinogenesis, metastasis, and drug resistance in various types of cancers^28–30^. Previously, it has been reported that ER stress could induce autophagy. When exposed to ER stress, misfolded protein accumulates, which augments the expression of proteins involved in the recovery process^31,32^. GRP78, as a critical component of this protective response, has been reported to be associated with autophagy. GRP78 plays a major role in regulating the activity of the macroautophagic receptor SQSTM1/p62 (sequestosome 1) via N-terminal arginylation by cellular stressors such as ROS and clearing the misfolded proteins in lysosomes via interaction with p62^28^. In addition, GRP78 negatively regulates the lysosomal activity through ubiquitination of MUL1; this phenomenon was closely related to the progression of head and neck cancer^16^. In the present study, we found that the expression of GRP78 was significantly higher in MDR GC cells than in the parental cells, and the knockdown of GRP78 significantly reversed MDR in GC. Moreover, GRP78-induced autophagy could enhance MDR in GC cells.

In summary, we demonstrated that miR-495-3p was downregulated in MDR cell lines and cancer tissues. In addition, the overexpression of miR-495-3p inhibited the GC MDR in vitro and in vivo by targeting GRP78 and regulating the autophagy process. Furthermore, the miR-495-3p/GRP78/mTOR axis provides an insight into the mechanisms underlying tumor drug resistance and may serve as a novel therapeutic target for the treatment of MDR in GC.

## Materials and methods

### Cell culture

The human gastric adenocarcinoma cell line SGC7901 and its MDR variants, SGC7901/ADR and SGC7901/VCR,14 were cultured in RPMI-1640 medium (Gibco, USA) supplemented with 10% fetal bovine serum (FBS) (ZETA, USA), 100 U/mL penicillin and 100 mg/mL streptomycin (Gibco, USA) at 37 °C in a humidified air atmosphere containing 5% CO_2_. Vincristine (VCR) and Adriamycin (ADR) (Selleck, USA) were added to the culture media of SGC7901/VCR and SGC7901/ADR cells at a final concentration of 1 and 0.5 mg/mL, respectively, to maintain the MDR phenotype.

### Samples acquisition

A total of 15 frozen paired GC patients’ specimens were selected. These patients had undergone surgical resection of primary gastric adenocarcinoma at the First Affiliated Hospital of the Fourth Military Medical University under the procedures approved by the Ethics Committee for the use of Human Samples of the Fourth Military Medical University (Xi’an, China).

### RNA extraction and real-time PCR (RT-PCR) analysis

Total RNA was extracted using TRIzol Reagent (Life Technology, USA). To detect the expression of miR-495-3p, RT-PCR of stem-loop was performed using miR-X miRNA first-strand synthesis kit (Takara Bio Inc, Japan). The specific primers, miR-495-3p and U6 were purchased from Takara. A SYBR Green PCR Kit (Takara Bio Inc, Japan) was used to determine the mRNA expression level of GRP78, ATG12, LC3B, BECLIN-1, ATG5, LAMP2, and FIP200. The β-actin level was used as an endogenous control and each sample was analyzed in triplicate.

### Transmission electron microscopy

SGC7901 and GC MDR cells were fixed in 2.5% glutaraldehyde at 4 °C, followed by fixation in 0.1 M sodium phosphate buffer at pH 7.4, 37 °C for 2 h. The tissues were dehydrated in an ethanol gradient and embedded for slicing ultrathin sections that were imaged using JEM-1230 (GEOL, Tokyo, Japan).

### Western blot, immunofluorescence microscopy and apoptosis assay

The immunofluorescence staining, western blot, and apoptosis assay were performed as described previously^15^. The antibodies used for the immunostaining were LC3B, P62, Atg12, Beclin-1, p-mTOR, mTOR, p-S6K, S6K, and p-4E-BP1 (Cell Signaling Technology, USA), GRP78 (Santa Cruz Biotechnology, USA), and p-62 (Abcam, UK).

### MTT assay

Briefly, 10^3^ cells were used for the assays in 200 μL complete medium that was added with the indicated concentration of chemotherapeutic drugs, and cultured for an additional 24 h. The cultures were assayed daily and absorbance measured at 490 nm on a microplate reader (Thermo Fisher, USA). Each experiment was performed in triplicate and repeated three times.

### Luciferase reporter assays

For the reporter gene assay, the cells were plated in 24-well plate and transfected with 0.5 μg GRP78 3′-UTR (untranslated region) luciferase reporter plasmids and GRP78 3′-UTR mutant1 and mutant2 luciferase reporter plasmids (Land biolog Co. Ltd, Guangzhou, China) using lipofectamine 2000 (Invitrogen, USA). The cells were also contransfected with the mimic nc or miR-495-3p mimic (50 nM) (Ribobio Co. Ltd, Guangzhou, China). The assays were performed 24 h after transfection using the dual luciferase reporter assay system (Promega Biotech Co, Ltd, USA). Firefly luciferase activities were normalized to Renilla luciferase activities. All experiments were performed in triplicate.

### In vivo tumorigenicity in BALB/C nude mice

BALB/C nude mice were bred and maintained in the Experimental Animal Center of the Fourth Military Medical University. 1 × 10^7^ SGC7901/ADR, transfected with lentivirus miR-495-3p and NC (GENECHEM, Shanghai, China), were injected subcutaneously into the right pad of the mice. Six mice were injected in each group and sacrificed 4 weeks after injection of GC cells.

### Immunohistochemistry and in situ hybridization (ISH)

The immunohistochemical staining of miR-495-3p and GRP78 was performed as described previously^33^. Two types of GC tissue microarrays were purchased from Superchip (Shanghai, China). Each array contained adjacent GC tissues and primary GC tissues from a total of 90 cases. ISH was performed using miR-495-3p probes from Exiqon (miRCURY LNA detection probe 5′- and 3′-DIG-labeled). The probe was detected using digoxigenin antibody (Abcam, UK), LSAB2 System-HRP (Dako Denmark A/S, Glostrup, Denmark), and DAB System (Dako, Denmark) according to the manufacturer’s instructions. The results of IHC and ISH were scored independently by two pathologists in a blinded manner. The scoring was based on the intensity and extent of staining. The staining intensity was graded as follows: 0, negative staining; 1, weak staining; 2, moderate staining; 3, strong staining. The proportion of stained cells per specimen was determined semi-quantitatively as follows: 0 for staining 0–1%; 1 for 1–25%; 2 for 26–50%; 3 for 51–75%; 4 for ≥75% of the examined cells. The histological score (H-score) for each specimen was computed by the following formula: *H*-score = Proportion score × Intensity score. A total score of 0–12 was graded into negative (−, score: 0), weak (+, score: 1–4), moderate (++, score: 5–8), or strong (+++, score: 9–12). Samples with *H*-scores > 4 were determined to have a high expression, and those with ≤4 were determined to have low expression.

### Statistical analysis

Statistical significance was calculated with GraphPad Prism Software using one-way ANOVA with Tukey’s post-test and Student’s *t*-test. Data are presented as mean ± SD. *P-values < 0.05, **P < 0.01, or ***P < 0.001 were considered as statistically significant.

## Electronic supplementary material

Supplementary figure 1

Supplementary figure 2

Supplementary figure 3

supplementary figure legends
