# Supplementary figures and images for: MicroRNA-495-3p inhibits multidrug resistance by modulating autophagy through GRP78/mTOR axis in gastric cancer
Source: Cell Death Dis. 2018 Oct 19;9(11):1070. doi: 10.1038/s41419-018-0950-x (PMC6195618; doi:10.1038/s41419-018-0950-x)

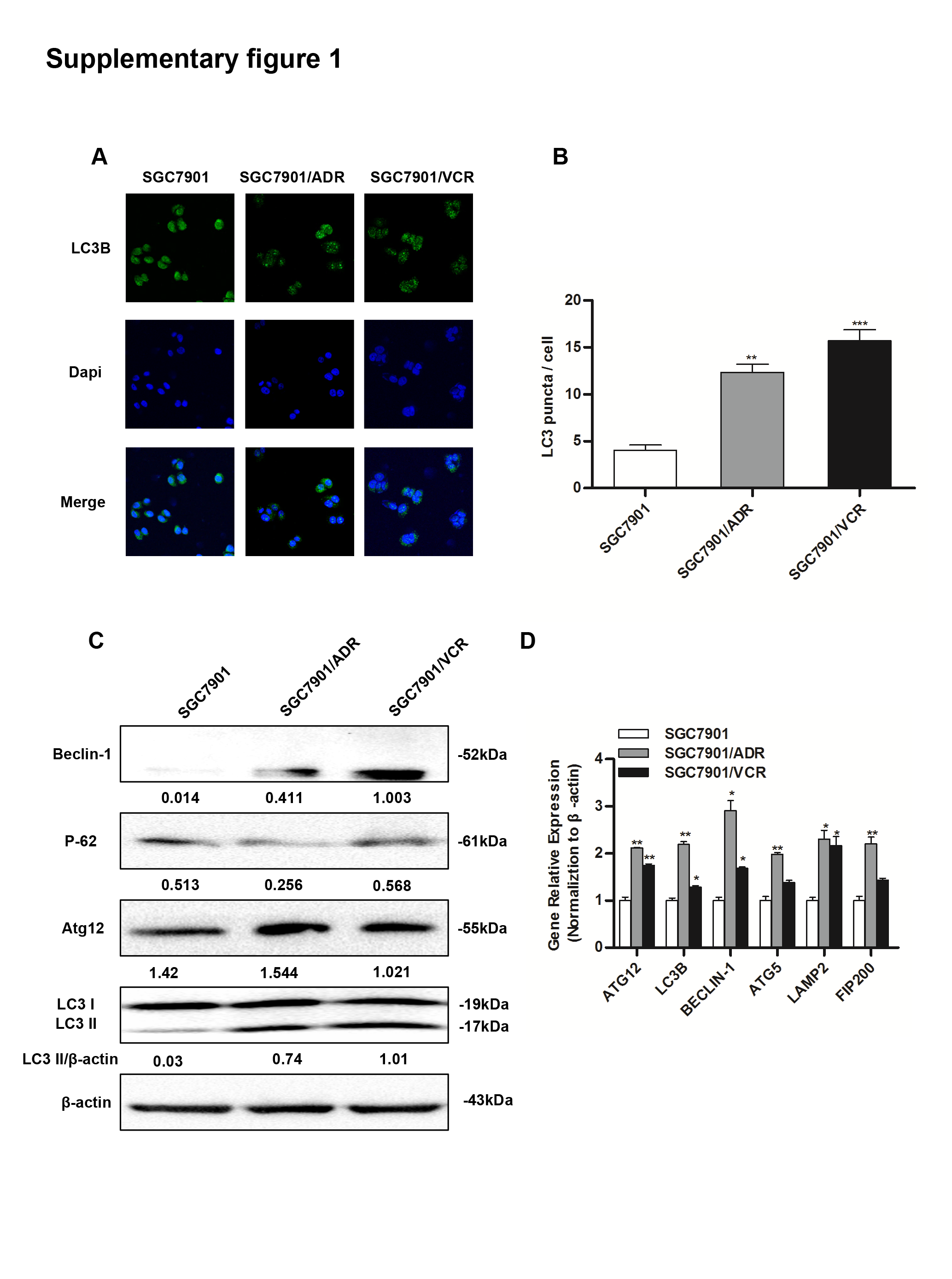

Supplement: Supplementary file 1 — Supplementary figure 1 [file 41419_2018_950_MOESM1_ESM.tif]

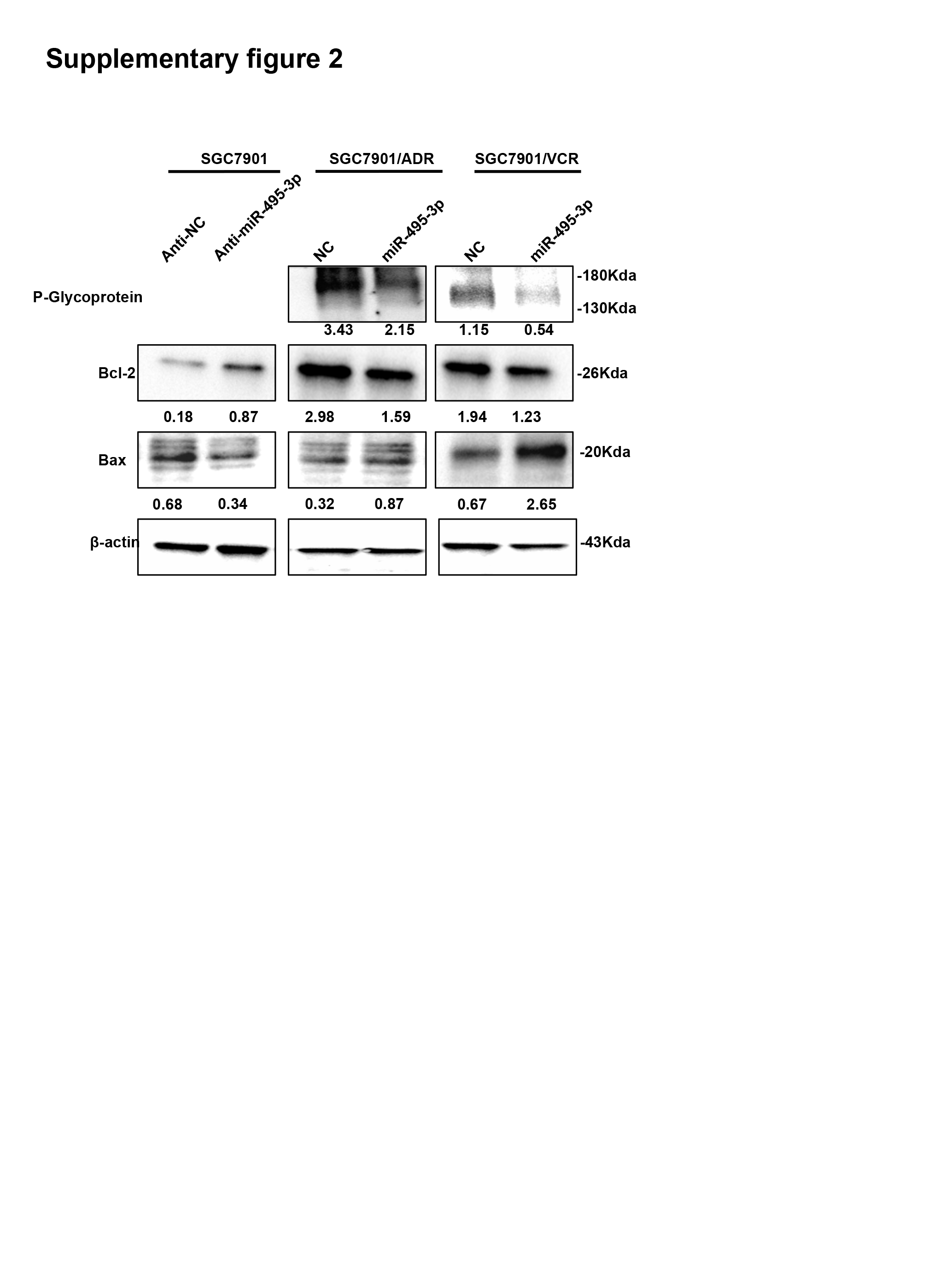

Supplement: Supplementary file 2 — Supplementary figure 2 [file 41419_2018_950_MOESM2_ESM.tif]

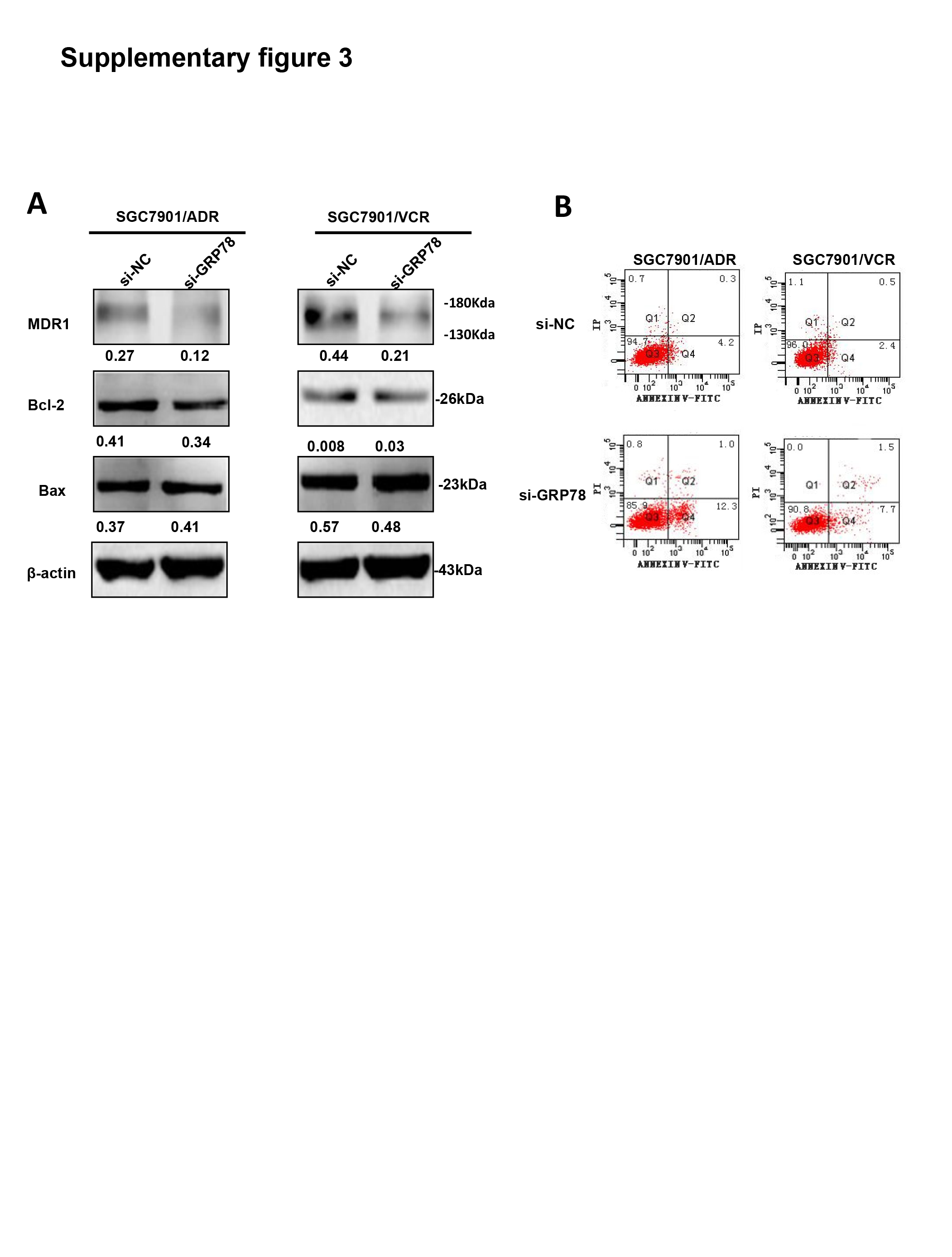

Supplement: Supplementary file 3 — Supplementary figure 3 [file 41419_2018_950_MOESM3_ESM.tif]
