# Supplementary material for: MicroRNA-495-3p inhibits multidrug resistance by modulating autophagy through GRP78/mTOR axis in gastric cancer
Source: Cell Death Dis. 2018 Oct 19;9(11):1070. doi: 10.1038/s41419-018-0950-x (PMC6195618; doi:10.1038/s41419-018-0950-x)
Supplement: Supplementary file 4 — supplementary figure legends [file 41419_2018_950_MOESM4_ESM.docx]

**Supplementary Figure 1 GC MDR cells show a high level of autophagy as compared to parental cells SGC7901.**

1. Representative images and (b) Quantification of LC3 puncta (green) in SGC7901 and GC MDR cells. (c) Western blot analysis of autophagy-associated protein including Beclin-1, P-62, Atg10, and LC3I/II in SGC7901 and its MDRs. (d) Real-time PCR determines autophagy-associated gene level in SGC7901 and its MDRs. All values expressed as mean ± SD, n = 3 for each group. *P < 0.05, **P < 0.01, ***P <0.001.

**Supplementary Figure 2 Upregulated expression of miR-495-3p inhibits P-glycoprotein and promotes apoptosis in GC MDR cells.**

Western blot analysis of P-glycoprotein, Bax and Bcl-2 in SGC7901 transfected with anti-miR-495-3p and GC MDR cells transfected with miR-495-3p.

**Supplementary Figure 3 Downregulated expression of GRP78 promotes apoptosis in GC MDR cells**

1. The levels of MDR, Bcl-2, and Bax were measured in GC MDR cells transfected with si-GRP78 and si-NC by Western blotting. (b) FACS analysis of PI^+^AnnexinV-FITC^+^ apoptotic cells in GC MDR cells after transfection with si-GRP78 and si-NC. n = 4 for each group.
